# *Fusarium* infection in malting barley drives trichothecene transfer and transformation during brewing process, impacting beer safety and composition

**DOI:** 10.1007/s12550-026-00649-x

**Published:** 2026-04-11

**Authors:** Eva Maria Biehl, Stefan A. Pieczonka, Ibrahim Can Akpak, Felix Hoheneder, Stefan Asam, Philippe Schmitt-Kopplin, Martin Zarnkow, Ralph Hückelhoven, Michael Rychlik

**Affiliations:** 1 Chair of Analytical Food Chemistry, TUM School of Life Sciences, Technical University of Munich, Freising, Germany; 2 Research Unit Analytical BioGeoChemistry, Helmholtz Munich, Neuherberg, Germany; 3 Chair of Phytopathology, TUM School of Life Sciences, Technical University of Munich, Freising, Germany; 4 Research Center Weihenstephan for Brewing and Food Quality, Technical University of Munich, Freising, Germany

**Keywords:** Mycotoxins, Deoxynivalenol, *Fusarium culmorum*, Brewing, Metabolomics

## Abstract

*Fusarium* contamination in malting barley poses a constant threat of mycotoxin exposure in brewing. However, the chemical transformations of trichothecenes toxins during processing and the broader impact of fungal contamination on beer quality are insufficiently understood. In this study, we inoculated barley grains with *Fusarium culmorum* to track the behavior of deoxynivalenol (DON) and its conjugates, deoxynivalenol-3-glucoside (DON-3G) and 3-acetyldeoxynivalenol (3-AcDON), through a controlled malting and brewing sequence. Quantitative LC-MS/MS and qPCR analyses revealed extensive fungal activity and toxin formation in malt, reaching 1,054 µg kg⁻¹ DON and 4,352 µg kg⁻¹ DON-3G, respectively. Mashing and lautering facilitated the release of matrix-bound toxins, whereas fermentation represented the key stage for biochemical transformation. During this phase, yeast metabolism markedly increased free DON through the hydrolysis of DON-3G and deacetylation of 3-AcDON. During maturation, a partial re-glycosylation of DON was observed. Despite toxin conversions, the final *Fusarium* infected batch (FIB) still contained substantial residues of the original toxins load in malt: 99% of DON, 50% of DON-3G, and 84% of 3-AcDON. *Fusarium* likewise affected chemical and sensory quality of beer: beer from infected kernels showed darker color, elevated soluble nitrogen, and a higher Thiobarbituric Acid Index, pointing toward intensified Maillard reaction activity. Untargeted FT-ICR-MS and LC-ToF-MS analysis confirmed infection-driven shifts in the beer’s molecular composition, revealing elevated Maillard-derived compounds and a distinct biochemical imprint of infection marked by fungal metabolites such as hydroxytrichodiene and host-defense conjugates including cinnamoyl-serotonin derivatives. Our findings show that *F. culmorum* infection affects both consumer safety of beer and the fundamental (bio)chemistry underlying its composition.

**Supplementary Information:**

The online version contains supplementary material available at 10.1007/s12550-026-00649-x.

## Introduction

Beer is one of the economically most significant fermented beverages worldwide, and its quality and safety are shaped by a tightly coordinated sequence of technological and biochemical processes. The beer industry is fundamentally dependent on the quality of its primary raw material: barley malt. The malting process – a controlled steeping, germination, and kilning of barley—is designed to activate the kernel’s “enzymatic toolbox” (Narziss and Back 2012; Díaz et al. 2022). This process develops the amylases and proteases necessary to break down starches and proteins into the fermentable sugars and free amino nitrogen (FAN) that yeast requires for optimal growth. However, the warm, moist, and nutrient-rich conditions that are ideal for barley germination are also a supportive environment for the proliferation of filamentous fungi, particularly pathogenic *Fusarium* species, creating an inherent process risk (Pascari et al. 2018, 2022; Biehl et al. 2024).

In barley, contamination typically originates in the field through *Fusarium* head blight (FHB), where infection of the developing grain results in the accumulation of diverse mycotoxins (Parry et al. 1995). The most relevant of these in brewing are the type B trichothecene deoxynivalenol (DON) and the mycoestrogen zearalenone (ZEN) (Wolf-Hall et al. 1999). The malting process itself acts as a bioreactor, fundamentally altering the toxin profile. While some hydrophilic toxins like DON can be partially leached during steeping, the germination phase triggers a *de novo* synthesis of DON by the fungus (Biehl et al. 2024). Simultaneously, the barley kernel, in a defense response, metabolizes the toxin by attaching a glucose molecule, creating the “modified” mycotoxin deoxynivalenol-3-glucoside (DON-3G) (Piacentini et al. 2019). These toxins are thermostable, surviving kilning and transferring from the malt into the liquid wort during mashing (Mastanjević et al. 2018; Pascari et al. 2022). Although fermentation can reduce the toxic load via yeast adsorption, significant concentrations of both DON and DON-3G can persist in the final beer (Jouany et al. 2004; Campagnollo et al. 2015; Piacentini et al. 2019). Large-scale surveys have demonstrated that beers worldwide can contain substantial levels of *Fusarium*-derived toxins: Varga et al. (2013) detected DON and DON-3G in up to 89 µg/L and 81 µg/L, respectively, in beers from 38 countries, with detection rates exceeding 75%. Similarly, Peters et al. (2017) reported combined DON and DON-3G concentrations ranging from 10 to 475 µg/L in over 400 of 1,000 analyzed beers—most of them craft beers, which commonly include unconventional ingredients and diverse brewing materials.

The impact of a *Fusarium* infestation extends beyond toxicological safety, severely compromising the technological and sensory quality of the beer. A primary defect is “gushing,” the spontaneous and uncontrolled over-foaming of beer upon opening (Niessen et al. 1982). This phenomenon appears directly linked to hydrophobins, highly surface-active proteins produced by the *Fusarium* fungi that survive the brewing process (Sarlin et al. 2007, 2012). Fungal contaminations also degrade the beer’s chemical matrix. Increased proteolytic activity during malting can lead to abnormally high FAN levels (Pascari et al. 2022). This excess of amino precursors intensifies Maillard reactions during kilning and boiling, resulting in a darker, non-standard beer color. Furthermore, the pathogenic stress in germinating kernels increases lipid peroxidation, reducing the beer’s oxidative stability and shortening its shelf-life, taste and sensory properties (Guido and Ferreira 2023).

To unravel the multifaceted impact of fungal grain infestation on beer chemistry, advanced metabolomic strategies are applied to map beer’s molecular complexity (Cavallini et al. 2021). This analytical framework spans from targeted methods, such as liquid chromatography-tandem mass spectrometry (LC-MS/MS) for the precise *quantification* of known compounds like mycotoxins, to untargeted, high-resolution mass spectrometry approaches. Techniques like Time-of-Flight mass spectrometry (ToF-MS) and Fourier Transform Ion Cyclotron Resonance Mass Spectrometry (FT-ICR-MS) provide a holistic *fingerprint*, encompassing thousands of previously uncharacterized molecules in beer (Pieczonka et al. 2020). While mycotoxin research has traditionally focused on tracking a limited number of known toxins, the broader molecular footprint of a *Fusarium* infection remains poorly characterized. In this work, we address this gap using controlled *Fusarium culmorum* inoculation to link mycotoxicology with metabolomics, aiming to capture both the established toxin pathways and the wider spectrum of fungal and host-derived metabolites that shape the chemistry of contaminated beer.

## Materials and methods

### Chemicals and standards

For this study, solvents, reagents, and analytical standards were sourced from various commercial suppliers (Supplementary Table S1). [^15^N_3_]-BEA, [^15^N_3_]-ENN A1, and [^13^C_4_]-T-2 were synthesized in our laboratory according to previously published protocols (Asam and Rychlik 2006; Hu and Rychlik 2012).

### Preparation of analytical stock solutions

The precise concentration and purity of most analytes (3-AcDON, 15-AcDON, BEA, DON, ENN A, ENN A1, ENN B, ENN B1, FUSX, HT-2, NIV, ZEN) and the in-house synthesized internal standards [^15^N_3_]-BEA, [^15^N_3_]-ENN A1, and [^13^C_4_]-T-2 were determined by quantitative NMR using methanol-d₄ as the solvent.

For compounds available in quantities too small for qNMR analysis, the concentration was determined based on the certified specifications provided by the manufacturer. This approach was used for DON–3G, [^13^C_17_]-3-AcDON, [^13^C_15_]-DON, [^13^C_21_]-DON-3G, and [^13^C_22_]-HT-2. Stock solutions for all unlabeled and labeled toxins were initially prepared at a concentration of 10 µg/mL. Acetonitrile was the solvent for DON, DON-3G, 3-AcDON, 15-AcDON, FUSX, HT-2, NIV, T-2, and ZEN, while methanol was used for BEA, ENN A, ENN A1, ENN B, and ENN B1. These stocks were subsequently diluted to create working solutions at final concentrations of 1, 0.1, and 0.01 µg/L. All prepared solutions were kept at 4 °C in the dark.

### Raw material

Spring barley of the cultivar Solist, harvested in 2019, served as the raw material for the malting experiments. The grains were obtained from IREKS GmbH (Kulmbach, Germany) and stored in jute bags in darkness at 4 °C until needed for experimentation. As reported in Biehl et al. (2024), this batch had previously been analyzed for its fungal load and was found to exhibit only low levels of contamination. The defined provenance, the provision of the grains as a single batch by an industrial malting company, the controlled storage conditions, and the confirmed low fungal contamination qualify this material as an appropriate and reliable control barley for the present study.

### Inoculation and malting process

The entire process, from inoculum preparation to final malting, was conducted according to the detailed protocol established by Biehl et al. (2024). The key steps are summarized below.

A spore suspension of *Fusarium culmorum* (isolate *Fc*002), originally isolated as a single-spore strain from barley grain collected in Bavaria (Germany), was prepared by cultivating the isolate on ¼ strength PDA media for 14 days at 21 °C. Barley grains were surface sanitized using a hydrogen peroxide (H_2_O_2_) solution before being inoculated with 2% (*v/w*) of the spore suspension with a final concentration of 4 × 10⁵ CFU/mL. A control batch was prepared using sterile tap water. The grains were then incubated for 5 days at 25 °C and 96% relative humidity (RH). The laboratory-scale malting process was based on the MEBAK standard method R-110.45.008 (MEBAK online 2016a). It included a three-day steeping process to a target moisture of 44.5%, a three-day germination at 14 °C and 98% RH, and a multi-step kilning profile culminating at 80 °C for 5 h. To create representative material, the final malts and green malts from replicate experimental runs were pooled and homogenized. To create a reference for the brewing experiment, a corresponding control sample was prepared by inoculating barley with sterile tap water. This resulted in a pair of final malt samples for comparison: a control batch (CB) and a *Fusarium culmorum*-infected batch (FIB).

### Brewing process

For the brewing trials, each malt batch was first milled into grist using a two-roller dry mill (Mini Compact SE, Bühler, Uzwil, Switzerland) with a set roller gap of 0.8 mm.

A 5.2 kg charge of grist was used for each brewing trial. The grist was mashed in with 18 L of pre-heated water (62 °C), and an initial rest was held at 62 °C for 5 min with continuous stirring. Subsequently, the temperature was raised to 65 °C (at a rate of 1 °C/min) for a 45-min maltose rest. A final saccharification rest was conducted at 73 °C for 45 min, or until a negative iodine test was achieved. The process concluded with a mash-out step at 78 °C for 2 min.

The mash was then transferred to a pre-heated lauter tun. Following a 10-min rest period, the wort was recirculated for 10 min until it ran clear, at which point the collection of the first wort began. After the first wort was collected, the spent grain bed was sparged sequentially with two batches of 8 L and one batch of 10 L of pre-heated water (78 °C) to wash out the remaining extract. The collected wort was transferred to the kettle and brought to a rolling boil (100 °C). At the start of the boil, 41 g of Tradition hop pellets (Type 90, 5.8% α-acids, 2019 harvest, Hopsteiner, Hallertau, Germany) were added to achieve a calculated bitterness of 17 IBU. The total boil time was 60 min at atmospheric pressure. At the end of the boil, the original gravity was adjusted to 11.5°P using distilled water, and the hot trub was removed via a whirlpool step. The wort was cooled to 8 °C using a counter-flow chiller, transferred to a fermentation tank, and pitched with 28.5 g of dry yeast (TUM 34/70, SafLager™, Fermentis, Belgium), corresponding to a pitching rate of 15 × 10⁶ cells/mL. Primary fermentation was carried out at 13 °C until the apparent extract reached a level of less than 2°P. A maturation phase followed at the same temperature until the diacetyl concentration was below 0.15 mg/L (as determined by GC). Subsequently, the beer was lagered for three weeks at 1 °C and then filled into 0.5 L brown glass bottles. The finished beer had an alcohol by volume of 5.3% and an apparent degree of fermentation of 81% (control batch) and 83% (*F. culmorum* infected batch).

### Sampling and sample preparation

The sampling and sample preparation procedures during malting followed the detailed methods published by Biehl et al. (2024). Briefly, samples (approx. 20–30 g) were collected at critical processing stages. Due to their high moisture content, samples of green malt and spent grains were freeze-dried for at least 48 h. The content of *Fc* DNA and various mycotoxins was determined in the starting barley, green malt, malt, and spent grain samples, according to the analytical protocols established for solid samples. For the isothermal mash, ground malt was extracted with water for 60 min, following the MEBAK standard method R-207.00.002, with a temperature of 20 °C instead of 65 °C, as described in the literature (MEBAK online 2016c). Liquid samples were collected at multiple stages of the brewing process, including grist, mash, spent grains, sweet wort, boiled wort, green beer, and finished beer. Approximately 0.5 L of each liquid sample was taken for analysis and further analyzed for mycotoxins.

### Quantification of genomic DNA with qPCR (barley and *F. culmorum*)

The extraction of genomic DNA from all samples and the subsequent quantification of *Fc* DNA relative to barley DNA were performed precisely as described in our previous work (Biehl et al. 2024). In brief, the method combines a modified DNA extraction protocol from the European Reference Laboratory for Genetically Modified Food and Feed (Joint Research Centre 2007; Linkmeyer et al. 2013), a qPCR assay using species-specific primers (Nicolaisen et al. 2009). Results were normalized and expressed as pg of *Fusarium* DNA per ng of barley DNA.

### Sample preparation for mycotoxin analyses

The preparation of solid samples for mycotoxin analysis was performed as described previously in Biehl et al. (2024). Briefly, the method involves an acetonitrile/water extraction of 1 g of ground sample, followed by a SPE clean-up using Bond Elut Mycotoxin (500 mg, 3 mL) (Agilent Technologies, Santa Clara, USA) cartridges.

The sample preparation of liquid samples (beer) was conducted based on the method by Habler et al. (2017) with several modifications. For extraction, performed in duplicate, a 2.5 mL aliquot of the degassed sample was spiked with internal standards, and 7.5 mL ACN was added. The mixture was agitated on a horizontal shaker (225 rpm, 15 min) and centrifuged (3200 x g, 5 min, 4 °C). The resulting pellet was then re-extracted twice with 1.5 mL of ACN/H₂O (70/30, *v/v*) each time. After the combined supernatants were evaporated to dryness at 40 °C, the resulting residue was redissolved in 4 mL of ACN/H₂O (84/16, *v/v)*. This solution was applied to a Bond Elut Mycotoxin SPE cartridge (500 mg, 3 mL) (Agilent Technologies, Santa Clara, USA) and the eluate was collected under a gentle vacuum. After a second evaporation step, the final residue was redissolved in 100 µL of ACN/H₂O (1/1, *v/v*) and membrane-filtered (PVDF, 0.22 μm). For quantitative analysis, SIDA was employed, with spiking levels adjusted after an initial qualitative screening of the samples. Samples were subsequently spiked with internal standards at a concentration appropriate for the linear range of the respective analyte’s response curve. Analytes without a corresponding labeled standard (NIV, and ZEN) were quantified using a matrix-matched calibration curve with four different amounts of analyte (15–75 µg/kg NIV and 0.04–0.32 µg/kg ZEN) prepared in a toxin-free beer matrix. In advance, toxin-free beer matrix was prepared by treating degassed beer with activated carbon at a concentration of 2.5 g/L. The suspension was agitated on a horizontal shaker (225 rpm, 15 min), centrifuged (3200 x g, 5 min), and the supernatant was subsequently clarified by membrane filtration (0.45 μm). Until LC-MS/MS analysis, samples were stored at 4 °C in the dark.

### LC-MS/MS mycotoxin quantification

Mycotoxin analysis was performed using a Shimadzu Nexera X2 UHPLC system coupled to a Shimadzu 8050 triple quadrupole mass spectrometer (Shimadzu, Kyoto, Japan). Chromatographic separation was achieved on a Shim-pack Velox PFPP column (10 × 2.1 mm, 2.7 μm) equipped with a matching guard column (5 × 2.1 mm, Shimadzu, Duisburg, Germany), with the column oven maintained at 30 °C.

Due to the different ionization properties of the analytes, two separate chromatographic methods using electrospray ionization (ESI) were developed. A negative ESI mode was used for DON, DON-3G, NIV, and ZEN, while a positive ESI mode was used for 3-AcDON, 15-AcDON, BEA, ENN A, ENN A1, ENN B, ENN B1, FUSX, HT-2, and T-2. For the negative mode, a gradient of water and acetonitrile was used. The negative ESI gradient, initially developed for the analysis of solid sample extracts, was modified for liquid matrices (beer and mash) to mitigate matrix effects that interfere with type B trichothecenes. The initial mobile phase condition was changed from 10% to 1% acetonitrile to improve chromatographic separation and resolve the analytes from interfering components. For the positive mode, the mobile phases consisted of 0.1% formic acid in water and 0.1% formic acid in methanol. Both methods utilized a flow rate of 0.4 mL/min and a sample injection volume of 4–5 µL with a co-injected water volume of 40–46 µL. The detailed gradient programs for both modes are provided in Supplementary Table S2.

The mass spectrometer was operated in the scheduled multiple reaction monitoring (MRM) mode. The specific ion source parameters, including temperatures, gas flows, and voltages for both positive and negative ESI modes, are listed in Supplementary Table S3.

The optimized MRM transitions (including precursor and product ions and collision energies) for each analyte are detailed in Supplementary Table S4. Data acquisition and processing were performed using LabSolutions software (Shimadzu, Kyoto, Japan).

### Calibration and quantitation

Quantification of mycotoxins was achieved using different calibration strategies. For DON, DON-3G, T-2, HT-2, 3-AcDON, ENN A1, and BEA, SIDA was employed using their respective isotopically labeled analogues. Calibration curves were constructed by plotting the peak area ratios of the analyte to the standard against their molar ratios over a range of 0.01–100. A surrogate internal standard approach was used for other analytes, where [^13^C_17_]-3-AcDON served as the standard for 15-AcDON, and [^15^N_3_]-ENN A1 was used for ENN A, ENN B, and ENN B1, with response curves generated in the same manner. For the remaining analytes (NIV, ZEN, and FUSX), which lacked an internal standard, an 11-point external matrix-matched calibration was performed. A mycotoxin-free potato starch and a toxin-free beer matrix were spiked with standards to cover concentration ranges of 7.5–750 µg/kg for NIV, 0.04–45 µg/kg for ZEN, and 5–500 µg/kg for FUSX. These standards underwent the complete sample extraction and clean-up procedure. To ensure data quality, three to four calibration points were analyzed with each sample batch to confirm the validity of the established curves. The linearity for all analytes was verified by Mandel’s fitting test (Mandel 1964).

### Method validation

Method validation for solid samples (barley, malt) was already described in Biehl et al. (2024).

The analytical method was validated for liquid samples by determining the limits of detection (LODs), limits of quantification (LOQs), precision, and recovery rates. LODs and LOQs were calculated according to the method of Vogelgesang and Hädrich (1998) using a toxin-free beer matrix spiked at four different concentration levels. Method precision was evaluated for *intra-day* (*n* = 3) and *inter-day* (*n* = 9 over three weeks) repeatability, while instrumental precision was assessed from ten consecutive injections of a standard mixture. Apparent recovery rates were determined by analyzing blank matrix samples spiked with each analyte at three concentration levels. All validation experiments were performed in triplicates.

### Additional process analyses

In the final beer samples, a range of attributes were measured according to standardized methods at the accredited laboratories of the Research Center Weihenstephan for Brewing and Food Quality in Freising. These analyses included the content of amino acids (Method LS-HPLC001 2018-07), free amino nitrogen (FAN) (MEBAK online 2020a), fermentable carbohydrates (MEBAK online 2020b), soluble nitrogen (MEBAK online 2020c), pH value (MEBAK online 2016b), the Thiobarbituric Acid Index (TBI) (MEBAK online 2020d) and the color value (MEBAK online 2023). These brewing attributes are used to characterize the final beer and are relevant for quality control throughout the brewing process.

### Metabolome analysis

#### FT-ICR-MS metabolite profiling

Due to their interfering nature in ESI direct infusion, the saccharides from the beer samples were separated using solid-phase extraction (SPE) (Supplementary Table S5). The eluted samples were directly injected into the solariX Fourier transform Ion Cyclotron Resonance mass spectrometer (FT-ICR-MS) at a flow rate of 2 µL/min (Bruker Daltonics GmbH, Bremen, Germany) equipped with a 12 T superconducting magnet (Magnex Scientific Inc., Yarton, GB) and a APOLO II ESI source (Bruker Daltonics GmbH, Bremen, Germany) operated in negative ionization mode (400 scans, *m/z* 120 to 1000) without any further dilution. Source and ICR cell parameters, data calibration, processing, and filtering followed established protocols from previous studies (Pieczonka et al. 2021). A total of 3,728 unique molecular formulas were assigned to monoisotopic mass using network calculations (Tziotis et al. 2011) within the CHNOSPCl compositional space, with an average mass error of ± 0.15 ppm, reproducibility in at least 2 of 3 replicates, and a signal-to-noise ratio exceeding 6.

#### LC-ToF-MS metabolomics

The SPE treated, undiluted beer samples were analyzed using a Sciex X500R QTOF system (AB Sciex, Darmstadt, Germany) in data dependent acquisition and positive ionization mode, coupled to a UPLC ExionLC (AB Sciex, Darmstadt, Germany) chromatograph. Detailed chromatographic and mass spectrometric parameters can be found in Supplementary Table S5. Resulting .wiff2 files were converted to mzML format using the MSConvert software (ProteoWizard) (Chambers et al. 2012). The parameters utilized for further data processing with mzMine 3 (Version 3.9.0) are outlined in Supplementary Table S6 (Schmid et al. 2023). The feature list was exported as an MFG file and subjected to analysis within the Sirius 5.8.2 software environment (Duhrkop et al. 2019). Tandem mass spectrometric data were employed to deduce molecular formulas using fragmentation trees (Bocker and Duhrkop 2016), characterize compound classes with CANOPUS (Djoumbou Feunang et al. 2016), and propose structural insights via CSI: FingerID (Duhrkop et al. 2015). The databases consulted and the parameters applied during this process are detailed in Supplementary Table S6. Identification confidence was defined as proposed by Schymanski et al. (2014), which involved outcomes from database searches, literature known references, as well as in silico calculated diagnostic ions and neutral losses (Clifford et al. 2006; Wu et al. 2009; Jaiswal et al. 2010; De Rosso et al. 2018). Reference standards (Supplementary Table S1) were utilized to achieve level 1 identification, whereby retention times and MS^2^ spectra were matched and confirmed via co-chromatography.

### Statistical data analysis and data visualization

#### OPLS-DA analysis

Following zero-filling, z-score normalization, and centering (Pieczonka et al. 2023), supervised OPLS-DA analysis were conducted to distinguish between the test and control brewing lines, executed with the ropls package (R 4.1.2) within the RStudio environment (version 2023.12.0). To mitigate the influence of substantial outliers on the models, we utilized Hotelling’s T2 test at a 95% data level. The accuracy and predictive capacity of the model were evaluated using the R2Y and Q^2^ metrics. Additionally, to guard against potential overfitting, the p-value from the Cross-Validation Analysis of Variance (CV-ANOVA) was computed. Significant features were determined by establishing a threshold for the Variable Importance for Projection (VIP) value, set at 2 for both FT-ICR-MS and LC-ToF-MS data, respectively.

#### Data visualization

The FT-ICR-MS compositional data and the significant molecular formulas derived from the OPLS-DA models were depicted in van Krevelen representations (hydrogen-to-carbon ratio against oxygen-to-carbon ratio) to identify compositional clusters and tentatively describe their respective (bio)chemical origin and compound classes (Schmitt-Kopplin et al. 2019).

## Results and discussion

### LC-MS/MS mycotoxin quantification

#### Sample preparation and validation

Analogous to the validation of the multi-mycotoxin analysis in barley and malt samples, described in Biehl et al. (2024), the validation for beer samples was carried out according to Vogelgesang and Hädrich (Vogelgesang and Hädrich 1998). The type B trichothecenes NIV, DON, DON-3G, 3-AcDON, 15-AcDON, and FUSX; the type A trichothecenes T-2 and HT-2; ZEN; as well as the enniatins A, A1, B, and B1 and BEA were determined in liquid matrices. To determine the LODs and LOQs, toxin-free beer was used as a blank matrix, spiked with the analytes at four different concentration levels, and processed in triplicate. LC–MS/MS measurements were likewise performed with triple injections. The contents of DON, DON-3, 3-AcDON, 15-AcDON, HT-2, T-2, ENN B, ENN B1, ENN A1, ENN A, and BEA were quantified using SIDA or IS, while NIV, ZEN, and FUSX were quantified by matrix calibration. The validation results are listed in Supplementary Table S7.

The multi-mycotoxin method applied to beer samples showed LODs between 0.002 µg/L (for ENN A and ENN A1) and 1.42 µg/L (for DON-3G) when analytes were quantified using SIDA or internal standards. Corresponding LOQs ranged from 0.005 µg/L (ENN A) to 5.02 µg/L (DON-3G). For analytes determined through matrix calibration, the LODs were higher, ranging from 0.016 to 3.72 µg/L, while LOQs extended from 0.054 to 11.0 µg/L. Among these, ZEN exhibited the highest sensitivity, with a quantification limit of 0.054 µg/L. Recovery experiments yielded results between 90% and 109%. The precision of the method was within 4% for instrumental measurements and up to 5% for *intra*- and *inter-day* analyses. Overall, the analytical procedures developed for both grain (Biehl et al. 2024), and beer samples proved to be sensitive, reproducible, and precise.

#### Mycotoxin and fungal DNA profile of malts for brewing trials

To generate quantifiable mycotoxin levels throughout the malting and brewing processes, barley was inoculated with a high concentration of *Fc* spores (4 × 10⁵ CFU/mL) to produce the infected malt. For this study, samples of the final infected malt and the corresponding green malt were created by pooling and homogenizing material from several replicate malting runs. The initial barley, the pooled green malt, the final malt, and the spent grains from the subsequent brewing process were analyzed for both mycotoxin content and *Fc* DNA. High-moisture samples (green malt and spent grains) were freeze-dried prior to analysis. To account for varying sample weights and dilution effects in liquid samples, mycotoxin results were also calculated as absolute amounts (µg).

Initial screening revealed that several mycotoxins, including T-2 toxin, FUSX, NIV, and ZEN, could not be quantified in either the control or the infected final malts. As these toxins were also not detected at any stage of the brewing process, they will not be discussed further.

The analysis of the control samples confirmed a very low basal fungal DNA contamination in the starting barley. After malting, no *Fc* DNA was detectable in the final control malt, indicating successful sanitation. The spent grains from the control brew showed only a negligible contamination level of 0.005 pg *Fc* DNA/ng barley DNA, confirming the suitability of this batch as a negative control. The control malt contained low background levels of DON at 5.84 ± 0.22 µg/kg and DON-3G at 9.56 ± 0.42 µg/kg. The acetylated derivatives, 3-AcDON and 15-AcDON, were not detected.

### Fate of toxins during the control brewing process

#### Trichothecenes

The behavior of DON and its modified form, DON-3G, showed contrasting trends during the brewing of the control beer (Table 1). DON was only found in very low amounts during the initial stages, with levels below the LOQ in both the mash and the spent grains. However, a significant increase in DON concentration was observed in the liquid fractions following the sweet wort. The final boiled wort contained 9.89 ± 0.41 µg/L DON, which further increased to 22.2 ± 2.23 µg/L in the young beer and remained stable at 22.5 ± 0.47 µg/L in the finished beer. 

**Table 1:** DON and DON-3G concentrations during the malting and brewing process of the control batch

Process steps	DON			DON-3G		
	[µg/kg] ([µg/L])	µg (absolute)	%	[µg/kg] ([µg/L])	µg (absolute)	%
barley	19.8 ± 0.48			6.58 ± 0.31		
green malt	n.d.			6.69 ± 0.10		
malt	5.84 ± 0.22	30.4	100	9.56 ± 0.45	49.7	100
mash	2.16*	38.9	-	2.51*	-	
spent grain	0.89*	-	-	2.86*	-	
sweet wort	6.40 ± 0.42	233	768	n.d.	n.d.	
boiled wort	9.89 ± 0.41	316	1041	n.d.	n.d.	
young beer	22.2 ± 2.23	642	2113	n.d.	n.d.	
beer	22.5 ± 0.47	607	1998	n.d.	n.d.	

n.d.=not detected

Values represent means of triplicate determinations ± SD. Absolute contents (µg) were calculated by multiplying the mean concentrations (µg/kg or µg/L) by the respective total amount. The percentage (%) indicates the ratio of each absolute content to the absolute content in the grist

In stark contrast, DON-3G, which was present in the malt, was only detectable at levels below the LOQ in the mash. After lautering, DON-3G was no longer detected in the sweet wort or any subsequent liquid brewing samples. This opposing behavior—the disappearance of the masked DON-3G concurrent with the appearance and increase of free DON—suggests the enzymatic hydrolysis of DON-3G during the mashing stage. Malt enzymes, likely β-glucosidases, may have cleaved the glucose moiety, releasing free DON into the wort, which was then carried through into the final beer.

The mash represents a complex matrix due to the high concentration of mono- and disaccharides, which can complicate the complete extraction of *Fusarium* toxins compared to pure grain flour. Consequently, higher standard deviations had to be tolerated for the mash samples, although these remained below 10% for all analytes. This increased matrix load may be the reason why the concentrations of DON and DON-3G in the control mash were below the LOQ, as the chromatograms exhibited elevated baseline noise. During the subsequent lautering step, the concentrations of these two toxins in the spent grains were also below the LOQ, indicating that no measurable quantities were removed from the process at this stage.

In the following liquid samples, only the aglycone DON was detected; DON-3G was no longer quantifiable from the sweet wort onwards.

#### Other mycotoxins

Other mycotoxins detected in the control batch were effectively removed from the liquid phases during brewing and were found to be preferentially partitioned into the solid fractions. As the inoculation was carried out with *Fusarium culmorum*, a known producer of type B trichothecenes, the subsequent evaluation is restricted to this toxin group. A comprehensive list of all toxin concentrations is presented in Supplementary Table S10.

### Mass balance of toxins during the brewing process

To evaluate the percentage change of mycotoxins during brewing, a mass balance was calculated. The absolute content (in µg) of each toxin in the initial malt grist was defined as 100%. The absolute content at each subsequent liquid processing stage was then calculated as a percentage of this initial value to determine any increase or decrease. All relevant process volumes, which are listed for both brewing trials in Supplementary Table S8 were factored into these calculations. Due to technical reasons, the spent grain samples were excluded from the mass balance analysis.

Of the mycotoxins analyzed, only DON could be quantitatively tracked throughout the entire brewing process. The mass balance for DON in the control brew is illustrated in Supplementary Figure S1 and listed in Table 1 for completeness.

It is important to note that due to the very low initial DON contamination in the control batch, even minor changes in the measured concentration resulted in large relative percentage shifts in the mass balance, than would be seen with higher contamination levels. No change was calculated for the mash, as the DON concentration was below the LOQ. A significant increase was first observed after the wort boil, with the absolute DON content in the boiled wort increasing by 273% compared to the sweet wort. The DON content increased further during fermentation and then remained stable throughout maturation and storage, with any minor fluctuations falling within the standard deviation of the measurements.

Overall, a total increase to 2,113% of the malt content in the absolute DON content was observed in the final control beer. However, it must be emphasized that the final concentrations in the beer were still comparably low.

An increase in the DON concentration was observed throughout the brewing process, whereas the maturation and storage of the beer did not further significant effect the toxin content. This increase in DON is consistent with the findings of Kostelanska et al. (2011b), who observed a 200–370% increase in DON content relative to the initial malt level during beer production. They attributed this to the possible release of matrix-bound toxins through enzymatic and physicochemical processes, particularly during mashing, which could also be the cause of the increase observed here.

The fact that DON-3G was no longer detected after lautering can also be explained by enzymatic processes. This could involve hydrolysis, degradation, modification to poly- and oligo-glucosides, or biotransformation into other DON adducts, such as DON-cysteine (Zachariasova et al. 2012; Kluger et al. 2015).

### Brewing with *F. culmorum* infected malt

#### Fungal DNA and mycotoxin profile of the *Fc* infected malt

The success of inoculation was confirmed by qPCR analysis, which showed a significant increase in fungal DNA in the green malt samples. The final brewing malt produced from this batch contained 2.77 pg of *Fc* DNA per ng of barley DNA. During the brewing process, the resulting spent grains still contained 1.05 pg *Fc* DNA/ng barley DNA. The concentrations of type B-trichothecenes in the starting materials and throughout the brewing process are shown in supplementary Table S11. As a result of the inoculation, the contaminated final malt contained a high load of mycotoxins: 1,054 ± 36.2 µg/kg of DON, 4,352 ± 127 µg/kg of DON-3G, 142 ± 3.89 µg/kg of 3-AcDON, and 10.1 ± 0.52 µg/kg of 15-AcDON (Supplementary Table S11).

#### Fate of mycotoxins during brewing with infected malt

The high initial mycotoxin load in the inoculated malt allowed for the quantitative tracking of their transformation throughout the brewing process (Table 2).

**Table 2:** DON, DON-3G, and 3-AcDON concentrations during the malting and brewing process of the *F. culmorum* infected batch

Process steps	DON			DON-3G				3-AcDON		
	[µg/kg] ([µg/L])	µg (absolute)	%		[µg/kg] ([µg/L]	µg (absolute)	%	[µg/kg] ([µg/L])	µg (absolute)	%
barley	19.8 ± 0.48				6.58 ± 0.31			n.d.		
green malt	472 ± 23.2				2,334 ± 32.4			100 ± 1.45		
malt	1,054 ± 36.2	5482	100		4,352 ± 127	22,629	100	142 ± 3.89	736	100
mash	140 ± 3.46	2519	46		626 ± 53.8	11,269	50	48.1 ± 1.35	866	118
spent grain	8.72 ± 0.07	-	-		27.5 ± 0.27	-	-	1.69 ± 0.01	-	
sweet wort	88.5 ± 2.57	3184	58		371 ± 50.6	13,342	59	31.3 ± 1.75	1125	153
boiled wort	94.6 ± 1.49	2933	54		381 ± 12.2	12,190	54	26.4 + 0.40	844	115
young beer	238 ± 10.6	6889	126		354 ± 30.8	10,260	45	24.5 ± 1.16	710	96
beer	201 ± 9.87	5432	99		417 ± 34.2	11,248	50	22.8 ± 0.35	616	84

n.d.=not detected

Values represent means of triplicate determinations ± SD. Absolute contents (µg) were calculated by multiplying the mean concentrations (µg/kg or µg/L) by the respective total amount. The percentage (%) indicates the ratio of each absolute content to the absolute content in the grist

#### Type B-trichothecenes

Following the mashing stage, the liquid mash contained 140 ± 3.46 µg/L of DON, 626 ± 53.8 µg/L of DON-3G, and 48.1 ± 1.35 µg/L of 3-AcDON. The concentrations of 15-AcDON, initially present in the malt at 10.1 µg/kg, were below the limit of quantification in all subsequent process samples and will not be discussed further. During lautering, the separation of the spent grains led to a decrease in the concentration of all toxins in the sweet wort (pre-hop addition) to 88.5 ± 2.57 µg/L for DON, 371 ± 50.6 µg/L for DON-3G, and 31.3 ± 1.75 µg/L for 3-AcDON. The spent grains themselves retained relatively low amounts of DON (8.72 ± 0.07 µg/kg), DON-3G (27.5 ± 0.27 µg/kg), and 3-AcDON (1.69 ± 0.01 µg/kg), with each representing approximately 1% of their original concentration in the malt grist. After the wort boil and removal of the hot trub, the concentrations in the boiled wort were 94.6 ± 1.49 µg/L for DON, 381 ± 12.2 µg/L for DON-3G, and 26.4 ± 0.40 µg/L for 3-AcDON, indicating general stability during this step.

The most significant transformations occurred during fermentation. The concentration of free DON more than doubled, rising to 238 ± 10.6 µg/L in the young beer, while the concentrations of DON-3G and 3-AcDON changed only marginally. This sharp increase in DON suggests active metabolic conversion by brewing yeast, likely through the hydrolysis of the modified mycotoxin DON-3G and deacetylation of 3-AcDON respectively and may also have been caused by the release of matrix-bound toxins (Zachariasova et al. 2012; Kluger et al. 2015). Interestingly, the subsequent maturation phase led to a reverse trend: the concentrations of DON and 3-AcDON decreased, while the concentration of DON-3G increased. The final beer contained 201 ± 9.87 µg/L of DON, 417 ± 34.2 µg/L of DON-3G, and 22.8 ± 0.35 µg/L of 3-AcDON. The increase in DON-3G during maturation could either indicate a detoxification mechanism by the yeast, involving the re-glycosylation of free DON or a cleavage of oligosaccharides (Table 2) (Zachariasova et al. 2012; Nathanail et al. 2016). Although other *Fusarium* toxins were also analyzed, the effects observed in this study can be attributed solely to the type B trichothecenes, as these additional toxins did not contribute to the transformations measured.

#### Other Mycotoxins

For completeness, other mycotoxins not produced by *F. culmorum* but present in the background were also analyzed and summarized in Supplementary Table S11.

#### Mass balance of mycotoxins in the *Fusarium culmorum* infected brew

To quantify the fate of the toxins, a percentage-based mass balance was calculated for the brewing process with inoculated malt. The absolute toxin content of the initial malt grist was set to 100%, and the content at each subsequent liquid stage was calculated relative to this value (Table 2, Supplementary Table S8). As with the control batch, the spent grain samples were excluded from this calculation for technical reasons.

**Fig. 1:**
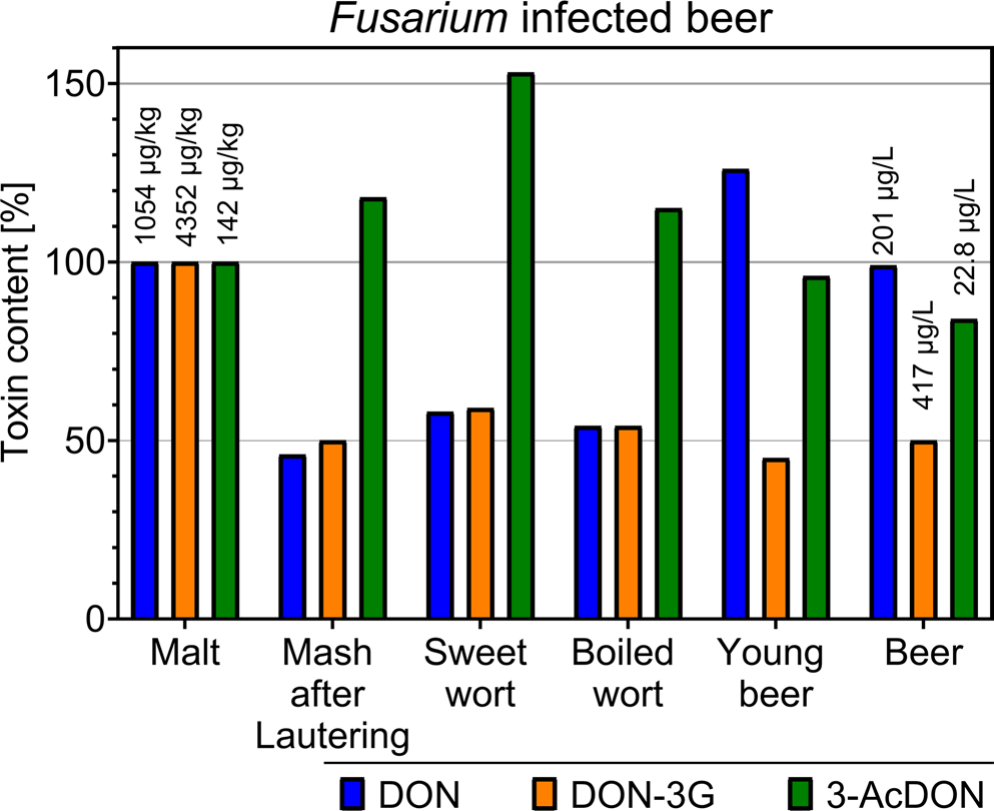
Mass balance of infected malt. Mass balance of the toxins DON, DON-3G, and 3-AcDON throughout the brewing process of the *Fusarium culmorum*-infected sample. The absolute toxin content of the malt grist was normalized to 100%, and relative changes in subsequent processing steps were calculated to visualize increases or decreases in toxin levels throughout the brewing process, toxin concentrations as well as brewing parameters are listed in Table 2 and in Supplementary Tables S8 and S11

The mass balance for the Type B-trichothecenes DON, DON-3G, and 3-AcDON is shown in Fig. 1 and listed in Table 2 for completeness.

The behavior of DON and DON-3G was similar up to the fermentation stage. In the mash, 46–50% of their initial content was recovered. This value then increased to 58–59% in the sweet wort after the lautering step. The wort boil did not significantly influence the concentrations of DON or its glucoside; any fluctuations observed in the pitched wort were within the standard deviation of the measurements.

Significant changes occurred during fermentation and maturation. The absolute content of DON increased during fermentation but then decreased during maturation, resulting in 99% of the original DON content from the malt being present in the final beer. Conversely, the DON-3G absolute content decreased during fermentation and was not further significantly affected by maturation, remaining constant between the young beer and final beer stages. Ultimately, 50% of the DON-3G content in the malt grist was transferred into the finished beer.

The behavior of 3-AcDON was notably different. Due to its lower overall concentration, the mass balance was highly sensitive to small fluctuations. Mashing led to an apparent increase in the 3-AcDON amount to 118% relative to the grist, suggesting potential release from bound forms or conversion from other derivatives. This increase continued through lautering. After cooling and trub removal, the content in the boiled wort was like the level after mashing. Fermentation and maturation then led to a decrease in 3-AcDON, with the final beer containing 84% of the amount originally present in the malt.

The contaminated malt exhibited very high concentrations of type B-trichothecenes, which was attributable to the targeted inoculation with *F. culmorum* prior to malting. In contrast to the control, the behavior of DON and its modified forms could be tracked quantitatively. Initially, DON and its glucoside DON-3G showed comparable behavior. After mashing, a decrease in their concentrations was observed, with only 46–50% of the initial malt content being recovered. In contrast, the relative amount of 3-AcDON increased to 118% in the mash. As with the control batch, an incomplete extraction and higher standard deviations (10–15%) were accepted for the mash samples due to their complex composition. Following lautering, an increase in the content of DON and DON-3G to 58–59% of the initial value was observed, which, as previously discussed, could be explained by the release of matrix-bound toxins (Kostelanska et al. 2011b).

These observed decreases in DON and DON-3G after mashing differ from some published results. Studies by Kostelanska et al. (2011a); Lancova et al. (2008) reported a 70–170% transfer of DON and an increase of DON-3G by up to 1,400% after mashing and lautering. In contrast, Habler et al. (2016) observed a decrease in DON concentration to 78–85% during mashing, while the DON-3G content increased to 120%. Our results regarding the stability of DON during the wort boil are consistent with the findings of Habler et al. (2016) and the known thermostability of these analytes (Schwarz et al. 1995; Lancova et al. 2008).

The most significant transformations occurred during fermentation. The absolute content of DON increased, whereas the concentrations of DON-3G and 3-AcDON decreased accordingly. Although the enzymatic hydrolysis of DON-3G and the deacetylation of 3-AcDON by yeast, may contribute to the observed increase in DON, the decrease in DON-3G and 3-AcDON accounted for only approximately one-third of the corresponding rise in DON. This indicates that additional DON-releasing mechanisms, such as the release of matrix-bound forms, must have been involved. The decreasing DON-3G concentration could be due to degradation to DON or the formation of other metabolites (Zachariasova et al. 2012; Kluger et al. 2015). In the final beer, 99% of the initial DON, 50% of the DON-3G, and 84% of the 3-AcDON from the malt were measured. These transfer rates are higher than those reported by Habler et al. (2016) (46% DON, 16% DON-3G, and 49% 3-AcDON), but the literature shows that the behavior of type B-trichothecenes during brewing is inconsistent. For example, transfer rates of approximately 70–80% of DON from malt to beer have been reported, while other studies observed increases in DON and DON-3G concentrations during brewing, likely due to enzymatic or physicochemical release of bound mycotoxins (Schwarz et al. 1995; Lancova et al. 2008; Kostelanska et al. 2011b).

### Correlation analysis and final quality of the beer from inoculated grain

A correlation analysis was performed between the *F. culmorum* DNA content and the concentrations of DON, DON-3G, and 3-AcDON in the solid samples (barley, green malt, malt, and spent grains). As shown in Table 3, a positive correlation was found for all toxins, which was statistically significant at a two-sided level of *p* ≤ 0.01, confirming the direct link between fungal growth and toxin production at high contamination levels.

**Table 3:** Pearson correlation coefficients between* F. culmorum* DNA and toxin concentrations of DON, DON-3G, 3-AcDON, and 15-AcDON during the malting and brewing process of the *F. culmorum*-infected samples

Malting temperature	Amount of spores	Mycotoxin			
		DON	DON-3G	3-AcDON	15-AcDON
14°C	4 x 10^5^ CFU/mL	0.904*	0.923*	0.939*	0.917*

The final beer produced from the *F. culmorum* infected batch exhibited distinct quality differences compared to the control. It had a noticeably darker color which was confirmed by a higher EBC value of 8.75. Furthermore, the total soluble nitrogen content (132 mg/100 mL) and the thiobarbituric acid index (TBI) (55.6 vs. 28.2) were also elevated compared to the control beer. These analytical results are summarized in the Supplementary Table S9. This indicate a clear impact of the fungal infection on the final beer’s characteristics, also reported in literature for less contaminated barley malt (200 µg/kg DON in malt) (Oliveira et al. 2012, 2013). Schwarz et al. (2002) have shown that *Fusarium* infection alters proteolysis during germination, either via microbial proteases or by modifying the expression of grain proteases. This results in shifts in protein fractions, elevated endoxylanase, β-glucanase, and proteinase activities, and consequently higher levels of soluble and free amino nitrogen, accompanied by darker color formation. Such generally increased hydrolase activity impacts beer quality and the higher abundance of reactive amino components contributes to intensified Maillard reactions during kilning (Geißinger et al. 2022). The fungus also increased the level of reactive oxygen species, leading to a higher TBI. These oxidative processes, combined with increased proteolysis (higher soluble nitrogen), intensify the Maillard reactions, which lead to the formation of color-intensive melanoidins that darken the beer, also reported in literature (Wolf-Hall et al. 1999; Schwarz et al. 2006).

### Risk assessment

From a risk assessment perspective, the control beer, with a DON content of 22 µg/L, likely poses a negligible health risk, as a 70 kg person would need to consume 3.2 L daily to exceed the group-TDI of 1 µg/kg body weight. Although the raw material used for the inoculated variant was experimentally subjected to a high level of *Fusarium* inoculation, it showed no visible signs of spoilage or quality defects, and the DON content in the resulting malt remained below the current regulatory maximum level, indicating that such material could still be processed in practice. In contrast, for the inoculated beer containing 201 µg/L of DON, a 70 kg person would only need to drink approximately 350 mL per day to reach the TDI for DON alone. When considering the tolerable sum of DON, 3-AcDON, 15-AcDON, and DON-3G, a 70 kg person would exceed the group-TDI by consuming just 140 mL of this beer per day. Notably, experimental studies have shown that the acetylated derivatives differ in their biological activity, with 15-AcDON generally exhibiting higher intestinal toxicity than 3-AcDON (Pinton et al. 2012). These calculations highlight the importance of including all relevant DON derivatives in future regulatory limits. Linkmeyer et al. (2016) report that DON is the most common trichothecene contaminant in Bavarian barley, with concentrations in winter barley often reaching or exceeding 200 µg/kg. Given that European regulatory maximum levels have historically focused on unprocessed cereals (e.g., a maximum level of 1000 µg/kg for DON in cereal grains under Commission Regulation (EC) No. 2024/1022), and considering that neither malting nor brewing reliably remove DON—and may even promote the formation or persistence of toxicologically relevant modified forms, the systematic monitoring of DON and its modified forms in malting barley, malt, and finished beers is scientifically warranted. The results from this study and the cited literature demonstrate that predicting the final content of modified mycotoxins like DON-3G based on the initial DON content is unreliable due to the high variability of their transformations. For a sound risk assessment, it is essential to establish specific maximum limits for both grain and beer that explicitly include the modified forms of DON.

### Changes in the beer’s molecular composition

In addition to analyzing beer quality attributes, toxin levels, their modified forms, transformations, and adsorptive removal, we investigated the effect of *Fusarium* infestation of barley raw materials on the general molecular composition of beer. To separate interfering saccharides, a solid-phase extraction (SPE) was performed (Supplementary Table S5).

### FT-ICR-MS molecular profiles

**Fig. 2:**
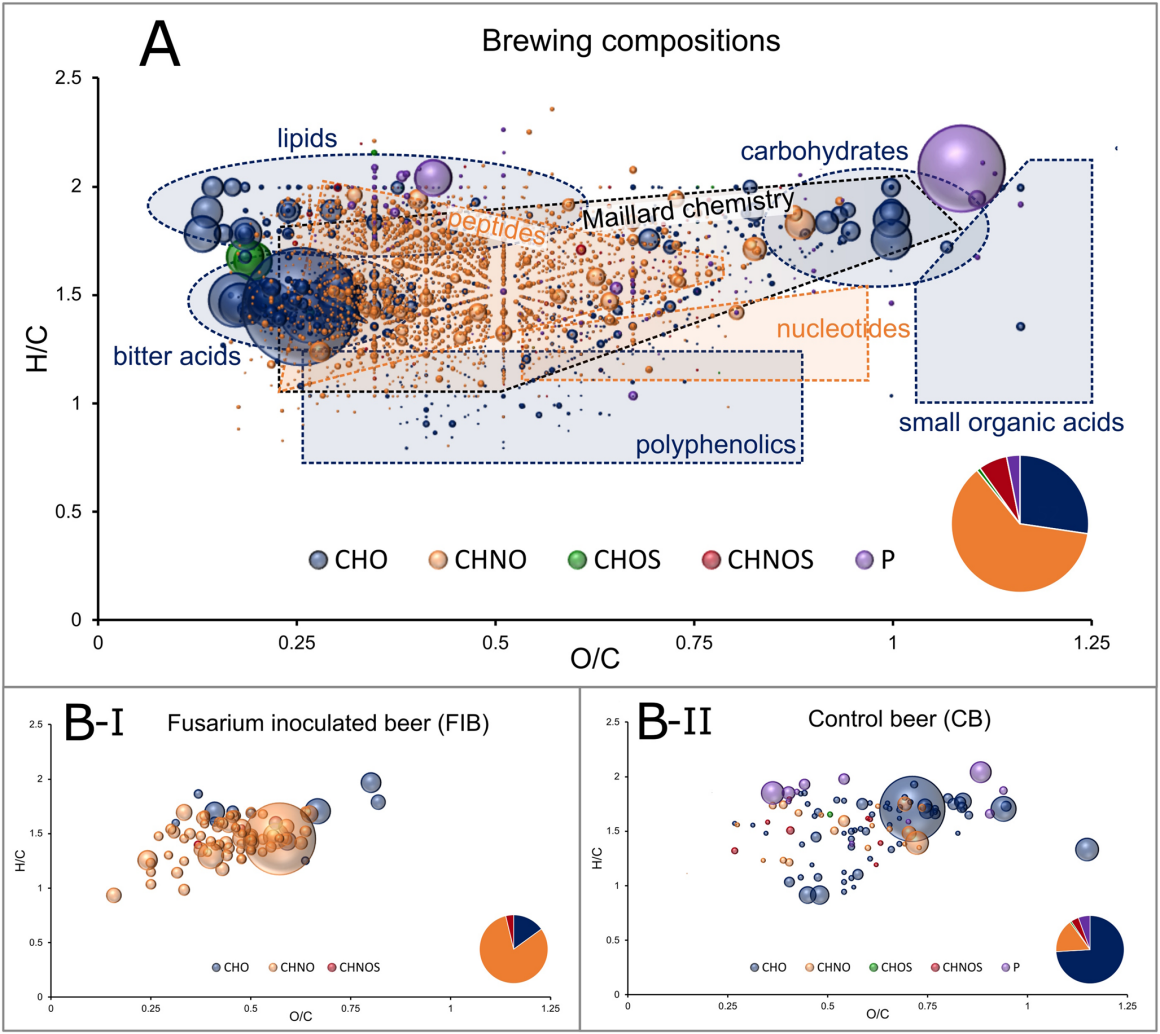
Van Krevelen representation of the DI-FT-ICR compositional data of the whole data set (**A**) and molecular profiles characteristic for the Fusarium infected (B-I) and control (B-II) beer. The intensities are represented as bubble sizes and scaled to the highest signal of the diagram. The color is defined by the compositional space (CHO: blue, CHNO: orange, CHOS: green, CHNOS: red, P-containing: purple) with their distribution indicated in pie charts. The molecular profiles in (**B**) were obtained by OPLS-DA analysis of all brewing line samples

Using the ultra-high resolving power of the Fourier Transform Ion Cyclotron Resonance Mass Spectrometer, we identified a total of 3,728 monoisotopic mass signals in the beer samples. The device’s mass accuracy (± 0.15 ppm) allowed for precise assignment of molecular formulas to these signals. This extensive molecular map provides a basis for characterizing beer metabolites based on their molecular composition (Pieczonka et al. 2020, 2022, 2023) (Fig. 2A).

This extensive molecular data set was analyzed using OPLS-DA statistics to identify compound signal profiles characteristic of brewing with *Fusarium*-infected barley. The differentiation of the two brewing lines in the predictive main component (Supplementary Figure S2A) captured the majority of the variance (R² = 0.99) between the y-variables *Fusarium*-infected (FIB) versus control (CB). The predictive power of the model was highly significant (Q² = 0.75). Consequently, the OPLS-DA model demonstrated that *Fusarium* infestation has a discernible impact on the beer’s metabolomic composition. The significant molecular profiles of the two brewing lines were defined using a Variable Importance for Projection (VIP) cut-off value of > 2 (Supplementary Figure S2B) and are presented in van Krevelen diagrams in Fig. 2B. Our molecular data support the assumption that darker beer coloration associated with fungal infection originate at the malting stage. Notably, the majority of the *Fusarium*-associated signals fall within the region of Maillard-derived products in the van Krevelen representation (Hemmler et al. 2018; Pieczonka et al. 2021, 2025) and around 45% correspond to molecular compositions previously linked to darker coloration in a large study of 250 beers (Pieczonka et al. 2021), while about 15% match peptide-derived formulas indicative of increased protein hydrolysis itself. Given the wide range of factors that shape beer composition, this degree of overlap must be considered substantial. Importantly, these compounds are seldom directly responsible for color, but rather reflect shifts in the underlying Maillard reaction, which ultimately leads to the formation of dark melanoidins. Consequently, a *Fusarium*-induced modulation of Maillard chemistry appears to be a major driver of the altered beer composition, though it is unlikely to represent the only mechanism involved.

### LC-ToF-MS metabolomics and compound identification

To obtain a complementary view of the beer metabolome, its alteration by fungal infection, and tandem mass spectrometric data, time-of-flight mass spectrometry (ToF-MS) analysis was conducted. A total of 3,211 chromatographic peaks were detected, reproducibly found in at least 2 out of 3 replicates. The OPLS-DA model of the ToF data showed a significant goodness of fit (R²Y = 0.98) and predictive power (Q² = 0.91) (Supplementary Figure S3A). The compounds that significantly contributed to the differentiation of brewing lines (Supplementary Figure S3B), and for which fragmentation spectra were obtained via Data Dependent Analysis (DDA), were further characterized using the Sirius software environment (Duhrkop et al. 2019) (Table 4). Their molecular formulas were determined through fragmentation trees and matched with the significantly accurate masses from FT-ICR-MS statistics (Bocker and Duhrkop 2016). The compound class was determined using the CANOPUS approach based on diagnostic ions and neutral losses (Djoumbou Feunang et al. 2016).

**Table 4:** Identification of compounds characteristic for the two brewing lines

Brewing line	VIP	m/z	retention [min]	formula (ToF)	formula(FT-ICR)	class	compound	Identification confidence
FIB	4.50	307.1447	5.80	C_19_H_18_N_2_O_2_	C_19_H_18_N_2_O_2_	cinnamic acid amide	cinnamoyl- serotonin derivative	level 2b
FIB	4.50	323.1398	5.31	C_19_H_18_N_2_O_3_	C_19_H_18_N_2_O_3_	cinnamic acid amide	Oxygenated cinnamoyl - serotonin derivative	level 2b
FIB	4.48	221.1903	6.58	C_15_H_24_O	-	sesquiterpene	hydroxy-trichodiene	level 2a
FIB	4.07	177.1026	2.81	C_10_H_12_N_2_O	C_10_H_12_N_2_O	tryptamine derivative	serotonin	level 1
CB	3.17	195.1383	5.85	C_12_H_18_O_2_	-	lipid lactone	-	level 3
CB	2.57	213.1488	6.15	C_12_H_20_O_3_	C_12_H_20_O_3_	jasmonic acid derivative	dihydrojasmonic acid	level 1
CB	2.06	190.0503	3.52	C_10_H_7_NO_3_	C_10_H_7_NO_3_	acridone alcaloid	kynurenic acid	level 1

m/z value, retention, molecular formula and compound class annotation lead to identification confidences of 1-3 following Schymanski et al. (2014)

The molecular formulas derived from the fragmentation spectra were consistent with accurate masses of significant FT-ICR-MS signals. Interestingly, the compounds showing the highest statistical significance in the complementary ToF measurements were not among those typically associated with Maillard reaction products. Their compound classes were assigned using CANOPUS and subsequently confirmed by further structural elucidation. Whenever available, proposed structures were validated against authentic reference substances. Serotonin, which likely derives from the plant´s *Fusarium*-triggered tryptophan metabolism (Hein et al. 2025), was identified as significantly upregulated in *Fusarium*-infected beers at a confidence level of 1 (Supplementary Figure S4A). By contrast, the tentative assignment of the cinnamic acid amide C_19_H_18_N_2_O_3_ as N-coumaroyl serotonin proved inaccurate upon comparison with a reference standard (Supplementary Figure S4D). Nonetheless, the substantial similarity of MS² spectra, despite differences in retention behavior, suggests structural relatedness. While N-coumaroyl serotonin shows a neutral loss of 176.0944 Da (serotonin, C_10_H_12_N_22_O) to the fragment *m/z* 147.0434 (coumaric acid, [C_9_H_7_O_2_]⁺), cleavage of the amide bond in compound C_19_H_18_N_2_O_3_ results in *m/z* 131.0498 (cinnamic acid, [C_9_H_7_O]⁺). The presence of an oxygenated serotonin moiety is therefore suggested, although it cannot be confirmed conclusively. Another infestation-associated phenylamine metabolite can be postulated as a deoxygenated variant of C_19_H_18_N_2_O_3_ with the formula C_19_H_18_N_2_O_2_. This compound contains the same cinnamic acid moiety as C_19_H_18_N_2_O_3_, as evidenced by the fragment ions at *m/z* 131.050, 103.055, and 77.039, along with the corresponding neutral loss of 130.0423 Da (C₉H₆O). The neutral loss of 176.0954 Da ([M + H]^+^ - C_10_H_12_N_2_O) yielding this cinnamic acid fragment, together with the fragment at *m/z* 177.1027 ([C_10_H_13_N_2_O]⁺), strongly indicates the presence of a serotonin moiety (Supplementary Figure S4). Together, these observations support its tentative assignment as cinnamoyl-serotonin. This is consistent with the recent report on convergent tryptophan and hydroxycinnamic acid metabolism in barley infected by *Fusarium culmorum* (Hein et al. 2025).

Conjugated tyramine and serotonin derivatives are produced by barley in response to biotic stress (Ishihara et al. 2017; Kurzweil et al. 2025). Ube et al. (2019a, 2019b)reported the antibacterial and antifungal activity of conjugated cinnamic acids (tryptamine derivatives) as phytoalexins in barley and wheat challenged with *Fusarium culmorum* or *Bipolaris sorokiniana*, respectively. Furthermore, we identified hydroxytrichodiene, a biosynthetic precursor of *Fusarium* trichothecenes in beer, by comparison with a published trichodiene fragmentation spectrum (Jelén et al. 1995) (Supplementary Figure S4E). On the control beer side, besides an undetermined fatty acid lactone (C_12_H_18_O_2_), dihydrojasmonic acid and kynurenic acid were identified as characteristic (Supplementary Figure S4B-C). Dihydrojasmonic acid is the reduced, biologically less active form of jasmonic acid, a phytohormone involved in plant development, signaling, and defense (Ghorbel et al. 2021). The accumulation of dihydrojasmonic acid, along with kynurenic acid as a tryptophan catabolite under normal metabolism, suggests that *Fusarium* infection triggers activation of dihydrojasmonic acid and associated defense pathways, shifting tryptophan metabolism toward phytoalexins such as the serotonin derivatives we identified. High-resolution metabolomics analyses, therefore, enable us to track the barley defense response throughout the brewing process and, ultimately, in the finished beer. Both fungal metabolites and alterations in plant metabolites contribute to the observed differences in the molecular composition of beer under *Fusarium* infection.

Our study demonstrates that *Fusarium culmorum* infection has consequences that extend beyond the presence of type B trichothecenes in malt. By following toxin behavior across the brewing process, we show that fermentation governs the major transformation steps, while subsequent maturation can further modify toxin conjugation patterns. Future non-targeted metabolomics studies focusing on enriched toxin extracts could provide deeper insight into transformations during the different processing steps. Importantly, contaminated malt consistently yielded beer with relevant trichothecene residues, underscoring the limited mitigating capacity of standard brewing operations. In addition to toxin persistence, the infection reshaped key chemical processes that underpin beer quality. Changes in color, nitrogen composition, oxidation markers, and the metabolomic profile collectively indicate that *Fusarium* infestation influences both fungal metabolite transfer and host-derived biochemical responses. Together, these findings highlight that fungal contamination of barley malt affects brewing on multiple levels, from mycotoxin dynamics to broader chemical and biochemical pathways, leaving a measurable imprint on the final beer’s safety, quality and composition. 

## Supplementary Information

Below is the link to the electronic supplementary material.

Supplementary Material 1 (PDF 1.71 MB)

## Data Availability

The FT-ICR-MS raw data will be made available by the authors upon reasonable request, without undue reservation. The LC-ToF-MS data set (mzML files) and the processed (mzMine3) mgf file exported to SIRIUS were uploaded to the MassIVE (UC San Diego) open access repository without access restrictions (Pieczonka 2025).

## Abbreviations

15-AcDON: 15-acetyldeoxynivalenol
3-AcDON: 3-acetyldeoxynivalenol
BEA: beauvericin
CFU: colony forming unit
DON: deoxynivalenol
DON-3G: deoxynivalenol-3-glucoside
ENN: enniatin
ESI: electrospray ionization
FUSX: fusarenone X
HT-2: HT-2 toxin
LC-MS/MS: liquid chromatography-tandem mass spectrometry
MEBAK: Mitteleuropäische Brautechnische Analysenkommission e.V.
NIV: nivalenol
qPCR: quantitative polymerase chain reaction
RH: relative humidity
RSD: relative standard deviation
SD: standard deviation
SIDA: stable isotope dilution assay
SPE: solid-phase extraction
T-2: T-2 toxin
ToF: Time-of-flight
ZEN: zearalenone
