# Supplementary material for: Fusarium infection in malting barley drives trichothecene transfer and transformation during brewing process, impacting beer safety and composition
Source: Mycotoxin Res. 2026 Apr 11;42(2):37. doi: 10.1007/s12550-026-00649-x (PMC13070047; doi:10.1007/s12550-026-00649-x)
Supplement: Supplementary file 1 — Supplementary Material 1 (PDF 1.71 MB) [file 12550_2026_649_MOESM1_ESM.pdf]

Supplementary Info

Tables

Table S1: Chemicals, reagents, and standards**.**

Chemical	Grade	Vendor
LC-MS/MS mycotoxin quantification		
Acetonitrile	analytical	Honeywell Riedel-de Haën (Seelze, Germany)
Methanol	analytical	Honeywell Riedel-de Haën (Seelze, Germany)
Formic acid	analytical	VWR (Darmstadt, Germany).
Hydrogen peroxide (30 %)	technical	VWR (Darmstadt, Germany).
Isopropanol	technical	VWR (Darmstadt, Germany).
Water	analytical	Th. Geyer (Renningen, Germany)
Potato starch	purified	Merck KGaA (Darmstadt, Germany)
DON	standard	Coring System Diagnostix (Gernsheim, Germany)
DON-3G	standard	Biopure (Tulln, Austria)
15-AcDON	standard	Biopure (Tulln, Austria)
3-AcDON	standard	Coring System Diagnostix (Gernsheim, Germany)
NIV	standard	Cayman Chemicals (Ann Arbor, USA)
T-2	standard	Biopure (Tulln, Austria)
HT-2	standard	Sigma Aldrich (Missouri, USA)
FUSX	standard	Coring System Diagnostix (Gernsheim, Germany)
ZEN	standard	Sigma Aldrich (Missouri, USA)
ENN A	standard	Cayman Chemicals (Ann Arbor, USA)
ENN A1	standard	Enzo Life Sciences (Lörrach, Germany)
ENN B	standard	Cayman Chemicals (Ann Arbor, USA)
ENN B1	standard	Enzo Life Sciences (Lörrach, Germany)
BEA	standard	AnaSpec (San Jose, USA)
$[^{13}C_{15}]$-DON*	standard	Biopure (Tulln, Austria)
$[^{13}C_{17}]$-3-AcDON*	standard	Biopure (Tulln, Austria)
$[^{13}C_{22}]$-HT-2*	standard	Biopure (Tulln, Austria)
$[^{13}C_{21}]$-DON-3G*	standard	Biopure (Tulln, Austria)
High-resolution mass spectrometry		
Acetonitrile	HiPerSolv MS-grade	VWR (Darmstadt, Germany)
Methanol	HiPerSolv MS-grade	VWR (Darmstadt, Germany)
Formic acid	HiPerSolv MS-grade	VWR (Darmstadt, Germany)
Water	ultrapure	Milli-Q Integral (Billerica, USA)
L-Arginine	analytical	Sigma Aldrich (St. Louis, USA)
LC-MS Tuning Mix	calibration	Agilent Techn. (Santa Clara, USA)

N-(p-Coumaroyl) Serotonin	reference standard	LGC Standards (Wesel, Germany)
(±)-9,10-Dihydrojasmonic Acid	reference standard	LGC Standards (Wesel, Germany)
Serotonin	reference standard	Sigma Aldrich (St. Louis, USA)
Kynurenic acid	reference standard	Santa Cruz Biotechn. (Heidelberg, Germany)

* Internal standards were bought as prepared solutions

Table S2: HPLC conditions and gradient programs.

Ionization and polarity	ESI negative
Flow rate	0.4 mL/min
Mobile Phase	A: H_2O<br>B: ACN
Oven temperature	30°C
Injection + co-injection volumes	5 µL Sample + 40 µL H_2O (solid samples)<br>4 µL Sample + 46 µL H_2O (liquid samples)
Gradient	

Solid samples (Barley, malt)		Liquid samples (Beer)	
Time [min]	Concentration B [%]	Time [min]	Concentration B [%]
0.00	10.0	0.50	1.00
2.00	10.0	1.00	10.0
6.00	99.0	7.00	99.0
7.50	99.0	8.50	99.0
9.00	10.0	10.0	1.00
11.0	10.0	13.0	1.00

Ionization and polarity	ESI positive
Flow rate	0.4 mL/min
Mobile Phase	A: H_2O + 0.1% Formic acid<br>B: MeOH + 0.1% Formic acid
Oven temperature	30°C
Injection + co-injection volumes	5 µL Sample + 40 µL H_2O
Gradient	

Time [min]	Concentration B [%]
0.00	6.00
2.00	6.00
16.0	90.0
18.0	99.0
19.5	99.0
21.0	6.00
23.0	6.00

Table S3: Mass Spectrometry Ion Source Parameters.

Analyte	NIV, DON, DON-3G, ZEN	FUSX, 3-AcDON, 15-AcDON, HT-2, T-2, ENN A, ENN A1, ENN B, ENN B1, BEA
Ionization and polarity	ESI negative	ESI positive
Interface Temperature [°C]	340	350
Heat Block Temperature [°C]	430	450
DL Temperature [°C]	170	150
Heating Gas Flow [L/min]	10	10
Drying Gas Flow [L/min]	10	10
Nebulizing Gas Flow [L/min]	1.4	3
CID Gas [kPa] (MRM)	230	265
Interface Voltage [kV]	- 4.5	3.0

Table S4: List of fragment ions and retention times (Rt) of the analyzed *Fusarium* toxins and their corresponding optimized collision energies (CE) and voltages.

Analyte	ESI +/-	Precursor ion *m/z*	Product ion *m/z*	Q1 pre-Bias [V]	Collision Energy [V]	Q3 pre-Bias [V]	Retention time [min]
NIV	-	311.20	281.20 [a]	20	13	30	1.07[s] / 1.30[l]
			138.20 [b]	20	24	30	
DON-3G	-	457.25	427.30 [a]	12	19	28	1.28[s] / 2.24[l]
			247.25 [b]	12	20	24	
[$^{13}C_{21}$]-DON-3G	-	478.25	447.30 [a]	12	19	28	1.28[s] / 2.24[l]
			261.25 [b]	12	20	24	
DON	-	295.30	265.20 [a]	10	14	10	1.47[s] / 2.14[l]
			247.20 [b]	10	15	40	
[$^{13}C_{15}$]-DON	-	310.30	279.20 [a]	10	14	10	1.47[s] / 2.14[l]
			261.20 [b]	10	15	10	
ZEN	-	317.15	175.10 [a]	24	25	16	5.19[s/l]
			131.05 [b]	24	30	22	
FUSX	+	355.10	175.20 [a]	-12	-22	-20	5.61
			137.20 [b]	-12	-26	-28	
15-AcDON	+	339.25	261.20 [a]	-10	-11	-30	7.62
			321.25 [b]	-10	-8	-6	
3-AcDON	+	339.10	231.25 [a]	-16	-13	-26	7.84
			175.20 [b]	-16	-25	-20	
[$^{13}C_{17}$]-3-AcDON	+	356.10	245.25 [a]	-16	-13	-26	7.84
			186.00 [b]	-16	-25	-20	
HT-2	+	447.15 [c]	345.15 [a]	-22	-19	-18	11.4
			285.20 [b]	-22	-21	-20	
[$^{13}C_{22}$]-HT-2	+	469.15 [c]	362.15 [a]	-22	-19	-18	11.4
			300.20 [b]	-22	-21	-20	
T-2	+	489.10 [c]	245.15 [a]	-26	-27	-29	12.6
			387.15 [b]	-14	-21	-22	
[$^{13}C_4$]-T-2	+	493.10 [c]	245.15 [a]	-26	-27	-29	12.6
			391.15 [b]	-14	-21	-22	
ENN B	+	640.75	196.25 [a]	-18	-25	-22	15.8
			214.25 [b]	-18	-25	-16	
ENN B1	+	654.30	196.25 [a]	-34	-26	-23	16.0
			210.25 [b]	-32	-24	-24	
ENN A1	+	668.70	210.25 [a]	-18	-24	-16	16.2
			100.20 [b]	-18	-60	-20	
ENN A	+	682.70	210.20 [a]	-12	-25	-16	16.4
			100.15 [b]	-12	-55	-20	
[$^{15}N_3$]-ENN A1	+	671.70	211.25 [a]	-18	-24	-16	16.2
			101.20 [b]	-18	-60	-20	
BEA	+	784.55 [c]	134.20 [a]	-22	-59	-26	16.3
			244.25 [b]	-22	-32	-28	
[$^{15}N_3$]-BEA	+	787.55 [c]	135.20 [a]	-22	-59	-26	16.3
			245.25 [b]	-22	-32	-28	

[a] Quantifier, [b] Qualifier, [c] Sodium adduct $[M+Na]^+$, [s] method for solid samples, [l] method for liquid samples

Table S5: SPE work-up, FT-ICR-MS and ToF-MS parameters.

Cartridge	Bond Elut PPL, 2 mL and 300 mg (Agilent Santa Clara, CA, USA)
conditioning	2,000 µL MeOH
	2 x 2,000 µL Milli-Q Water + 0.1 % FA
sample	2,000 µL acidified sample (0.1 % FA)
washing	1,000 µL Milli-Q Water + 0.1 % FA
dry vacuum	
elution	2 x 1,000 µL MeOH
FT-ICR Mass spectrometry	
sample preparation	SPE, see above
direct infusion flowrate	120 $\mu L.h^{-1}$
ESI capillary voltage	3600 V
time domain	4 mega words
accumulation time	0.25 ms
mass range	*m/z* 120 to 1000
accumulated scans	400
measurement time	10 min.
external calibration	clusters of arginine (5 $mg.L^{-1}$ in methanol)
internal calibration	in-house calibration list containing 2000 molecular formulae, which are highly abundant in beers (found in 33% of about 500 beers measured over the past years; data not shown)
LC-ToF-Chromatography	UPLC ExionLC (AB Sciex, Darmstadt, Germany)
sample preparation	SPE, see above
column	RP (C18: 1.7 µm, 2.1 x 100 mm, Acquity™ UPLC BEH™)
flow rate	400 µL min^{-1}
column oven temperature	40 °C
injection volume	5 µL (partial loop)
gradient profile	95 % A (0.1 % formic acid in water) and 5 % B (0.1 % formic acid in acetonitrile) for 1 min; decreasing to 0.5 % A in 9 min; held for 2.5 min; equilibrated in starting conditions for 1.5 min.
measurement time	15 min.
LC-ToF Mass spectrometry	X500R QTOF system (AB Sciex, Darmstadt, Germany)
external calibration	ESI positive calibration solution (SCIEX X500B System)
ESI ionization mode	positive
Ion source gas 1|2	45 | 45 psi
Curtain gas	30 psi
interface temperature	500°C
interface voltage	4 kV
MS^1 parameters	1.055 sec^{-1} event cycle time
	150-1500 Da mass range

	Accumulation time 0.2 sec
	Declustering potential 80 V
	Collision energy 5 eV
MS^2 fragmentation parameters	DDA (8 dependent events)
	Accumulation time 0.1 sec
	Time bins to sum 4
	20-1500 Da mass range
	CE spread 25 eV ± 15 eV

Table S6: Parameters of the UHPLC-ToF-MS data processing using the mzMine3 software and Sirius processing settings.

Parameter	value
**mzMine3**	
MS1 | MS2 noise level	300 | 10
Minimum peak height	1800
Minimum peak width	5 scans
m/z tolerance	0.005 Da or 10 ppm
Smoothing	Savitzky Golay (7)
Local minimum resolution	chrom. threshold 0.9
	Min ratio peak top / edge 2.0
13C isotope filter	applied
Peak alignment	0.001 Da or 10 ppm
	9 sec. RT tolerance
	m/z weight 3 | RT weight 2
MFG export (Sirius): MS/MS merge	Merge over all samples (MS1 0.005 Da)
	0.01 Da or 20 ppm
	Cosine threshold 0.6
	Signal count threshold 34 %
**Sirius**	
Sirius Molecular formula identification	$C_{\infty}H_{\infty}$ N_{∞} O_{∞} S_3 P_3
	MS^2 Mass accuracy 10 ppm
CSI:FingerID Fingerprint Prediction	$[M+H]^+$
CSI:FingerID Structure Database Search	Bio Database, Biocyc, CHEBI, COONUT, EcoCyc Mine, GNPS, HMDB, HSDB, KEGG, KEGG Mine, KNApSAcK, Maconda, MESH, NORMAN, Natural Products, Plantcyc, PubChem, PubMed, YMDB, YMDB Mine, ZINC
CANOPUS Compound Class Prediction	Main class: class (e.g. cinnamic acid amides)

Table S7: Validation data including limits of detection (LODs), limits of quantitation (LOQs), precision (RSD), and recoveries (3 different concentration levels) for 14 *Fusarium* toxins in beer. Recovery values of each spiking level were calculated as the mean value of three replicates and three injections. RSD = relative standard deviation; SIDA = stable isotope dilution assay; IS = internal standard quantification; MMC = matrix-matched calibration

Analyte	Analysis	LOD [µg/kg]	LOQ [µg/kg]	Precision (RSD) [%]			Recovery [%]*		
				inter-injection (n=10)	*intra-day* (n=3)	*inter-day* (n=9)	Level 1	Level 2	Level 3
DON	SIDA	1.17	4.32	2	1	3	100 ± 3	101 ±1	98 ± 5
DON-3G	SIDA	1.42	5.02	3	1	2	107 ± 3	100 ± 2	100 ± 1
3-AcDON	SIDA	0.52	2.40	3	2	3	94 ± 4	99 ± 4	106 ± 4
15-AcDON	SIDA	0.84	3.02	4	1	3	105 ± 3	100 ± 1	104 ± 1
HT-2	SIDA	0.42	1.79	3	1	1	109 ± 8	100 ± 1	101 ± 1
T-2	SIDA	0.25	1.12	2	1	1	103 ± 1	101 ± 0.4	100 ± 0.4
ENN A	IS	0.002	0.005	3	2	4	102 ± 2	101 ± 1	106 ± 1
ENN A1	SIDA	0.002	0.009	3	4	5	100 ± 5	100 ± 2	100 ± 1
ENN B	IS	0.005	0.014	3	2	1	97 ± 2	99 ± 2	93 ± 3
ENN B1	IS	0.009	0.04	3	3	2	100 ± 1	99 ± 0,2	95 ± 5
BEA	SIDA	0.002	0.006	3	2	1	102 ± 2	98 ± 3	105 ± 3
NIV	MMC	3.72	11.0	3	2	2	99 ± 1	98 ± 4	99 ± 3
ZEN	MMC	0.016	0.054	3	1	5	97 ± 6	95 ± 1	101 ± 1
FUSX	MMC	2.26	7.78	4	5	5	94 ± 2	90 ± 2	90 ± 2

* Spiking levels (Level 1, level 2, level 3) [µg/kg] of beer samples used for recovery determination were as follows: **DON** (7.5, 15, 25); **DON-3Glc (1**5, 40, 65), **3-AcDON (3,** 6, 10), **15-AcDON:** (8, 15, 20), **NIV (**15, 30, 50), **FUSX (**10, 20, 35), **ZEN (**0.03, 0.075, 0.15), **T-2 (**3, 7, 12), **HT-2:** (3, 7, 12), **ENN A** (0.03, 0.06, 0.3), **ENN A1** (0.005, 0.01, 0.02), **ENN B** **(**0.003, 0.01, 0.015), **ENN B1** (0.1, 0.25, 0.5) and **BEA** (0.015, 0.03, 0.06).

Table S8: Sample quantity and sampling points for balance calculation.

sample	control beer	*F. culmorum* infected beer
malt	5.2 kg	5.2 kg
mash	18 L	18 L
sweet wort	36.5 L	36 L
boiled wort	32 L	32 L
young beer	29 L	29 L
beer	27 L	27 L

Table S9: Analytical Results of the Finished Beers (after maturation), Determined by the Accredited Laboratory of the Research Center Weihenstephan for Brewing and Food Quality.

parameter	control beer	*F. culmorum* infected beer
original extract [°P]	12.1	11.8
alcohol [Vol.%]	5.27	5.25
real extract [°P]	4.05	3.88
real Degree of Fermentation [%]	66.4	67.3
pH value	4.59	4.61
color (according to EBC)	6.25	8.75
Thiobarbituric index (TBI)	28.2	55.6
total Soluble Nitrogen	116	132
total Free Amino Acids [mg/100 mL]	277	297
maltose [g/L]	0.40	0.30

Table S10: Mycotoxin concentrations during the malting and brewing process of the control batch. Values represent means of triplicate determinations ± SD. To calculate the absolute amounts (µg), the mean concentrations (µg/kg or µg/L) were multiplied by the corresponding total quantity. The percentage values (%) indicate the ratio of each absolute amount to that of the grist (reference). Mycotoxins not listed in the table were not detected in any sample. B = barley; M = malt.

Process step	Fc DNA pg/ng B. DNA	DON			DON-3G			HT-2			BEA		
		[µg/kg] ([µg/L])	µg (absolute)	%	[µg/kg] ([µg/L])	µg (absolute)	%	[µg/kg] ([µg/L])	µg (absolute)	%	[µg/kg] ([µg/L])	µg (absolute)	%
Barley	0.005	19.8 ± 0.48			6.58 ± 0.31			6.48 ± 0.22			0.60 ± 0.01		
Green malt	0.002	*n. d.*			6.69 ± 0.10			0.83 ± 0.04			0.40 ± 0.01		
Malt/Grist	0	5.84 ± 0.22	30.4	100	9.56 ± 0.45	49.7	100	1.28 ± 0.05			0.49 ± 0.03	100	
Mash	-	*< LOQ*	38.9	-	*< LOQ*	-		*< LOQ*	-		*< LOQ*	-	
Spent grains	0.005	*< LOQ*			9.25 ± 0.63			*< LOQ*			0.49 ± 0.02		
Sweet wort	-	6.40 ± 0.42	233	768	*n. d.*	-		*< LOQ*	-		*< LOQ*	-	
Boiled wort	-	9.89 ± 0.41	316	1041	*n. d.*	-		*< LOQ*	-		*< LOQ*	-	
Young beer	-	22.2 ± 2.23	642	2113	*n. d.*	-		*< LOQ*	-		*< LOQ*	-	
Beer	-	22.5 ± 0.47	607	1998	*n. d.*	-		*< LOQ*	-		*< LOQ*	-	

Process step	ENN A			ENN A1			ENN B			ENN B1		
	[µg/kg] ([µg/L])	µg (absolute)	%	[µg/kg] ([µg/L])	µg (absolute)	%	[µg/kg] ([µg/L])	µg (absolute)	%	[µg/kg] ([µg/L])	µg (absolute)	%
Barley	0.08 ± 0.00			0.49 ± 0.02			4.74 ± 0.40			1.94 ± 0.17		
Green malt	0.12 ± 0.02			0.39 ± 0.01			1.43 ± 0.07			0.97 ± 0.08		
Malt/Grist	0.10 ± 0.01	0.53	100	0.44 ± 0.00	2.30	100	2.30 ± 0.14	12.0	100	1.59 ± 0.11	8.2	100
Mash	*n. d.*	-		0.010 ± 0.00	0.18	8	0.15 ± 0.00	2.74	23	*< LOQ*	-	
Spent grains	*< LOQ*			1.21 ± 0.03			19.3 ± 0.60					
Sweet wort	*n. d.*	-		0.013	0.48	21	0.03 ± 0.00	1.21	10	*< LOQ*	-	
Boiled wort	*n. d.*	-		*< LOQ*	-		0.04 ± 0.00	1.21	10	*< LOQ*	-	
Young beer	*n. d.*	-		*< LOQ*	-		*< LOQ*	-		*< LOQ*	-	
Beer	*n. d.*	-		*< LOQ*	-		*< LOQ*	-		*< LOQ*	-	

n.d. = not detected

Table S11: Mycotoxin concentrations during the malting and brewing process of the *F. culmorum*–infected batch. Values represent means of triplicate determinations ± SD. To calculate the absolute amounts (µg), the mean concentrations (µg/kg or µg/L) were multiplied by the corresponding total quantity. The percentage values (%) indicate the ratio of each absolute amount to that of the grist (reference). Mycotoxins not listed in the table were not detected in any sample. B = barley; M = malt.

Process steps	Fc DNA pg/ng B. DNA	DON			DON-3Glc			3-AcDON			15-AcDON			HT-2		
		[µg/kg] ([µg/L])	µg (absolute)	%	[µg/kg] ([µg/L]	µg (absolute)	%	[µg/kg] ([µg/L])	µg (absolute)	%	[µg/kg] ([µg/L])	µg (absolute)	%	[µg/kg] ([µg/L])	µg (absolute)	%
Barley	0.005	19.8 ± 0.48			6.58 ± 0.31			*n. d.*			*n. d.*			6.48 ± 0.22		
Green malt	2.069	472 ± 23.2			2,334 ± 32.4			100 ± 1.45			4.35 ± 0.17			1.76 ± 0.13		
Malt/Grist	2.771	1,054 ± 36.2	5482	100	4,352 ± 127	22,629	100	142 ± 3.89	736	100	10.1 ± 0.52			1.70 ± 0.12		
Mash	-	140 ± 3.46	2519	46	626 ± 53.8	11,269	50	48.1 ± 1.35	866	118	< *LOQ*	-		< *LOQ*	-	
Spent grains	1.046	8.72 ± 0.07			27.5 ± 0.27			1.69 ± 0.01	-		< *LOQ*			< *LOQ*		
Sweet wort	-	88.5 ± 2.57	3184	58	371 ± 50.6	13,342	59	31.3 ± 1.75	1,125	153	< *LOQ*	-		< *LOQ*	-	
Boiled wort	-	94.6 ± 1.49	2933	54	381 ± 12.2	12,190	54	26.4 + 0.40	844	115	< *LOQ*	-		< *LOQ*	-	
Young beer	-	238 ± 10.6	6889	126	354 ± 30.8	10,260	45	24.5 ± 1.16	710	96	< *LOQ*	-		< *LOQ*	-	
Beer	-	201 ± 9.87	5432	99	417 ± 34.2	11,248	50	22.8 ± 0.35	626	84	< *LOQ*	-		< *LOQ*	-	

Process steps	BEA			ENN A			ENN A1			ENN B			ENN B1		
	[µg/kg] ([µg/L])	µg (absolute)	%	[µg/kg] ([µg/L]	µg (absolute)	%	[µg/kg] ([µg/L])	µg (absolute)	%	[µg/kg] ([µg/L])	µg (absolute)	%	[µg/kg] ([µg/L])	µg (absolute)	%
Barley	0.60 ± 0.01			0.08 ± 0.00			0.49 ± 0.02			4.74 ± 0.40			1.94 ± 0.17		
Green malt	0.29 ± 0.02			0.06 ± 0.00			0.39 ± 0.01			1.56 ± 0.03			1.51 ± 0.06		
Malt/Grist	1.77 ± 0.04	9.19	100	0.09 ± 0.01	0.53	100	0.44 ± 0.00	2.31	100	4.15 ± 0.01	21.6	100	2.12 ± 0.09	11.0	100
Mash	< *LOQ*	-		*n. d.*	-		< *LOQ*	-		0.06 ± 0.00	1.13	5	< *LOQ*	-	
Spent grains	0.65 ± 0.02			< *LOQ*			1.72 ± 0.02			13.9 ± 0.78			5.73 ± 0.01		
Sweet wort	< *LOQ*	-		*n. d.*	-		< *LOQ*	-		0.09 ± 0.01	3.25	15	< *LOQ*	-	
Boiled wort	< *LOQ*	-		*n. d.*	-		< *LOQ*	-		0.02 ± 0.00	0.69	3	< *LOQ*	-	
Young beer	< *LOQ*	-		*n. d.*	-		< *LOQ*	-		< *LOQ*	-		< *LOQ*	-	
Beer	< *LOQ*	-		*n. d.*	-		< *LOQ*	-		< *LOQ*	-		< *LOQ*	-	

n.d. = not detected

Figures

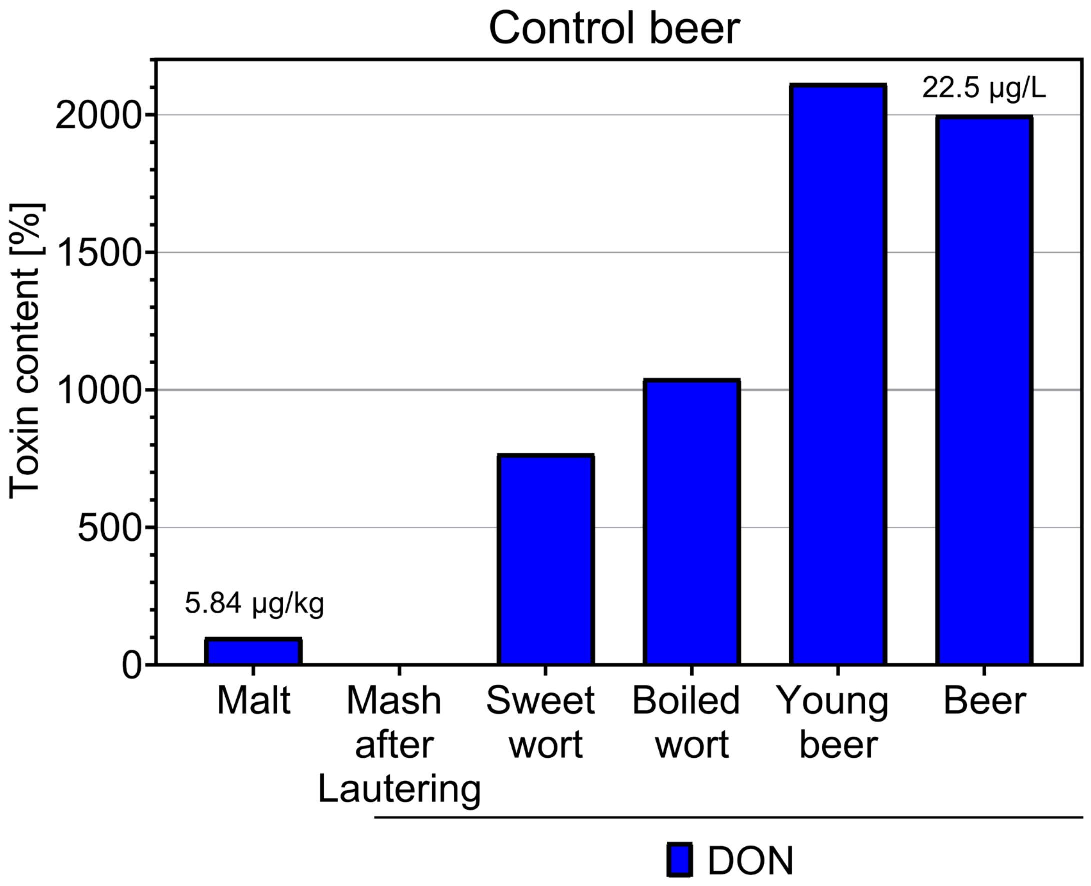

Figure S1: Balance of DON content during the brewing process of the control sample. The absolute toxin content of the malt grist was normalized to 100%, and relative changes in subsequent processing steps were calculated to visualize increases or decreases in DON levels throughout the brewing process, toxin concentrations as well as brewing parameters are listed in Table 1 and in Supplementary Tables S8 and S10. In the mash sample, the DON content was below the limit of quantification (LOQ) and therefore not included in the balance. Because DON levels were low throughout the brewing process in the control sample, even small absolute variations resulted in substantial percentage changes in the mass balance, highlighting the difference compared to the higher toxin concentrations observed in the *Fusarium*-infected batch.

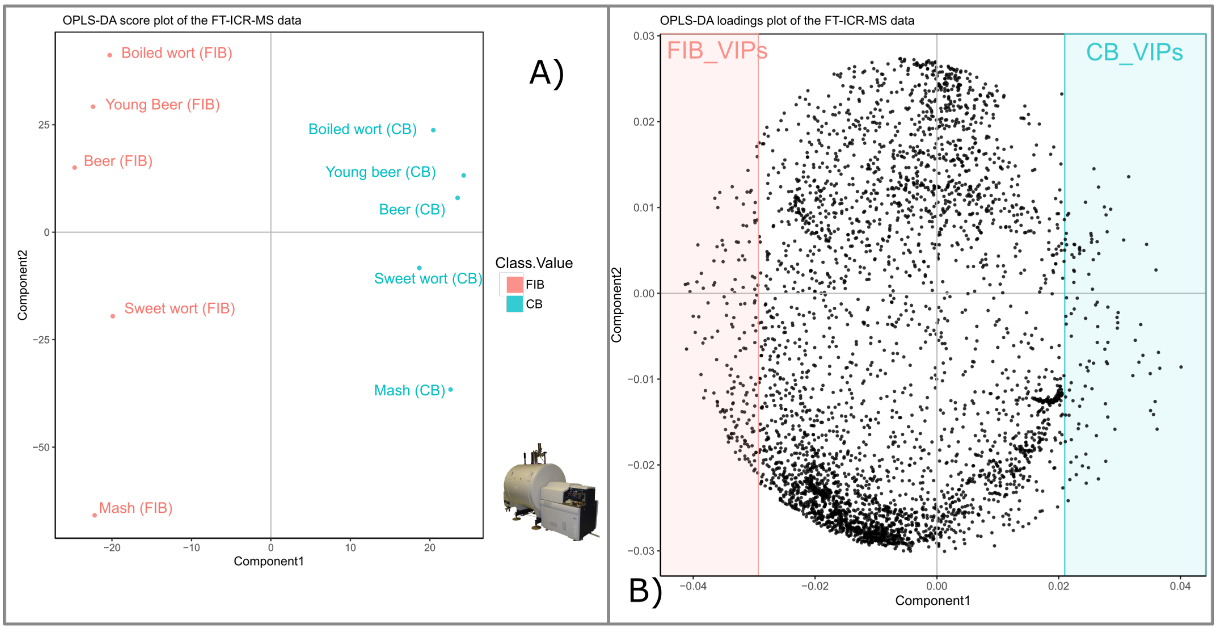

Figure S2: OPLS-DA score (A) and loadings plot (B) of the FT-ICR-MS data differentiating the *Fusarium* infected versus control brewing line. A Variable Importance in Projection (VIP) value cutoff of 2 was chosen (B).

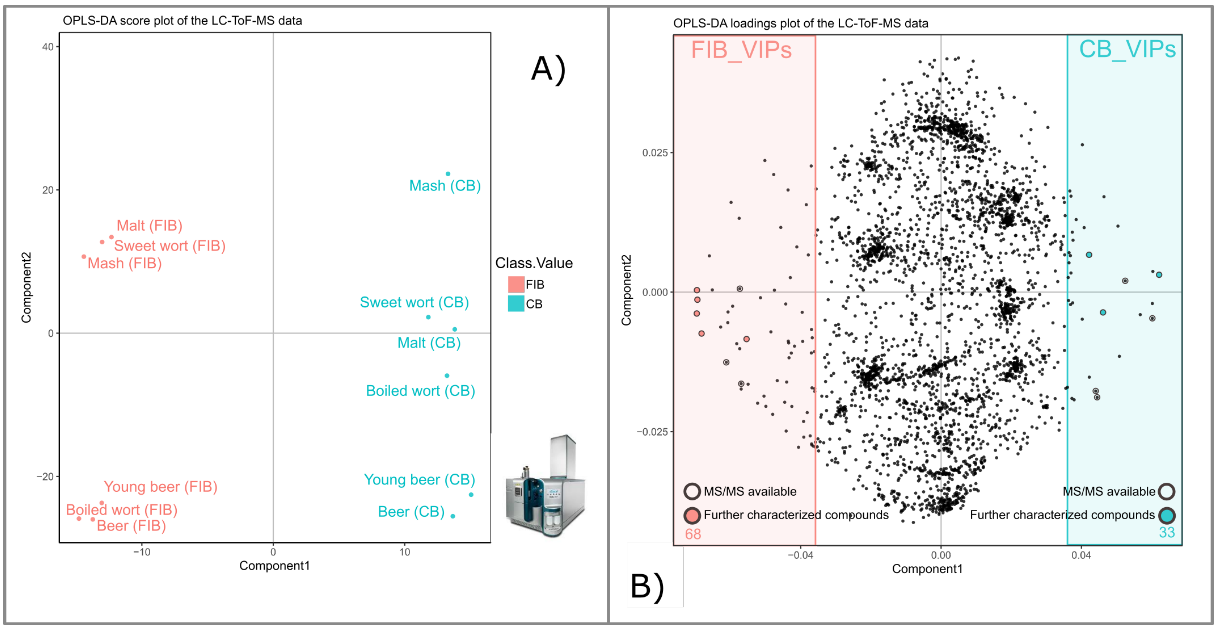

Figure S3: OPLS-DA score plot (A) and loadings plot (B) of the LC-ToF-MS data differentiating the *Fusarium* infected versus control brewing line. A Variable Importance in Projection (VIP) value cutoff of 2 was chosen (B). Identified compounds and features with MS^2 spectra are highlighted (up to the last identified compound).

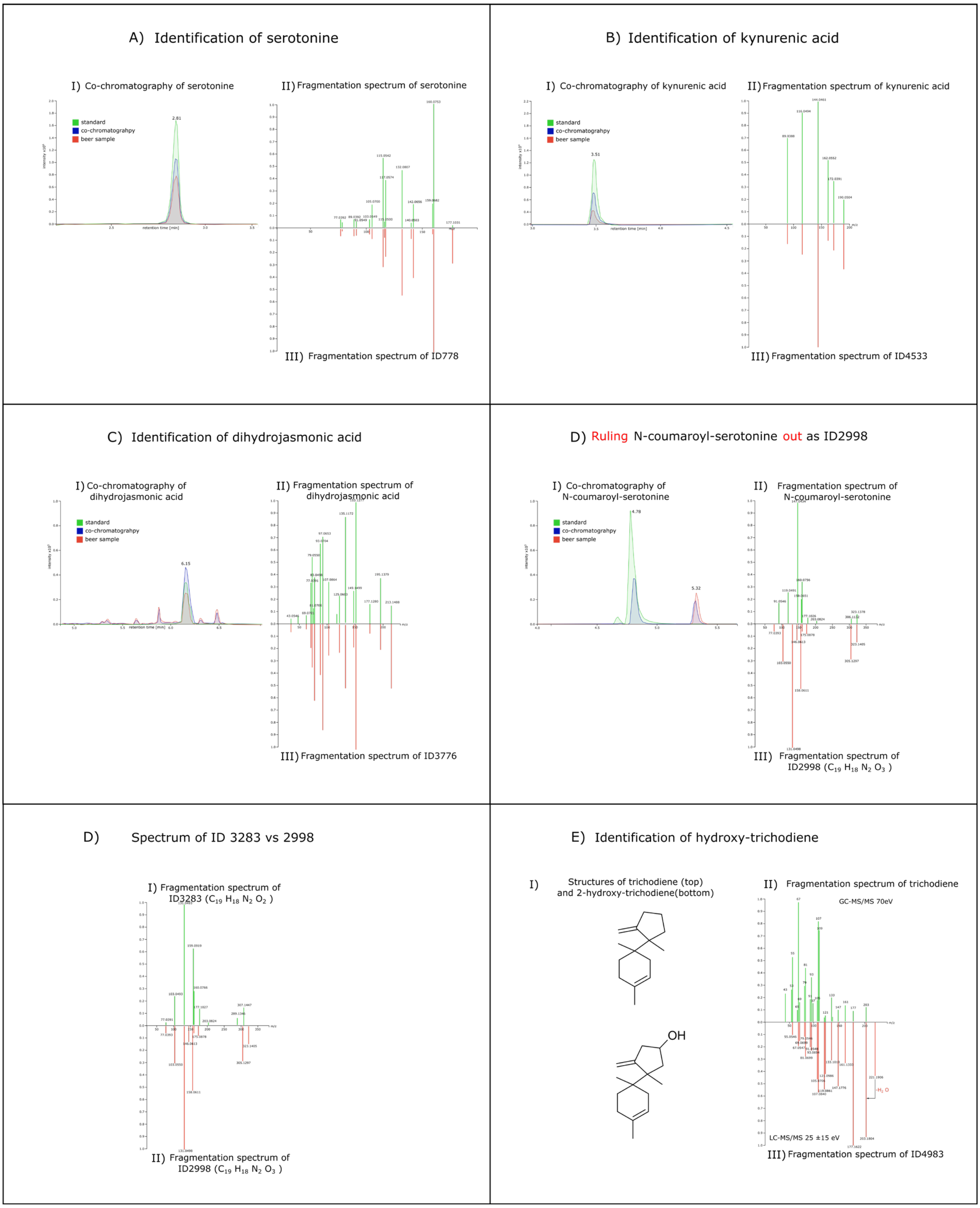

Figure S4: Identification of serotonine (A), kynurenic acid (B), and dihydrojasmonic acid (C); Ruling N-coumaroyl-serotonine out as ID 2998 (D), structural similarity of ID 3283 and 2998, and identification of hydroxytrichodiene via comparison against published MS^2 data of trichodiene on confidence level 2 (F). The compounds were identified through matching accurate masses and retentions (I) as well as fragmentation spectra (II-III). Compound 323.1398 | 5.31 min did not turn out to be N-coumaroyl-serotonin. Hydroxytrichodiene was identified by comparing it with the characteristic ions of trichodiene.
